# The implementation and preliminary evaluation of an ART strategy in Mexico - a country example

**DOI:** 10.1590/S1678-77572009000700019

**Published:** 2009

**Authors:** Vera Heriberto HERMOSILLO, Luengas Elisa QUINTERO, Namihira Delia GUERRERO, Díaz Dante Sergio SUÁREZ, Muñúzuri Jorge Alejandro HERNÁNDEZ, Christopher J. HOLMGREN

**Affiliations:** 1 DDS, Assistant Director of Oral Health, Ministry of Health, Mexico.; 2 DDS, Dentist attached to the Department of Oral Health of the Ministry of Health, Mexico.; 3 MSc, Research Branch of Teaching, Research and Training, Health Services of the State of Veracruz, Mexico.; 4 MSc, Professor, Division of Studies and Research, Faculty of Dentistry, Universidad Nacional Autonoma de Mexico, Mexico City, Mexico.; 5 DDS, Head of the Department of Prevention and Surveillance of Dental Fluorosis, Ministry of Health, Mexico.; 6 BDS, FDSRCS, PhD, Visiting Professor, Department of Global Oral Health, College of Dental Sciences, Radboud University Nijmegen Medical Centre, The Netherlands.

**Keywords:** Mexico, Health policy, Dental caries, Atraumatic Restorative Treatment (ART), Glass ionomer cements, Dental restoration, Pit and fissure sealants

## Abstract

The massive use of preventive measures in Mexico including fluoride toothpaste, a national program of salt fluoridation and education on prevention has resulted in a large decline in dental caries over the past two decades. There does however remain a largely unmet need for restorative treatment. This paper describes the steps leading up to the adoption of a strategy, as part of general health policy, to use Atraumatic Restorative Treatment (ART) within the Mexican public health service as a means to address this. This included the development of training materials, the organization of training courses for existing dentists and the incorporation of ART into the undergraduate curriculum. Results: Six years after the introduction of ART in the year 2000, it was estimated that over 2 million ART procedures had been provided. As part of the planning cycle, an evaluation was undertaken in 2008 to determine amongst Mexican dentists what were the perceived problems when implementing the ART approach. Such research identified that the scarcity of appropriate dental materials and the lack of suitable instruments were the major problems. In addition, a preliminary evaluation of ART restorations and sealants placed as part of this National Oral Health Program was undertaken. The survival outcomes after one year compared favorably with one other study conducted in Mexico but were somewhat lower than the results reported from a number of other countries. Conclusion: The ambitious and forward thinking policy for improving the oral health in Mexico is now showing dividends. One example is the ART strategy, which has been successful both in terms of the number of ART procedures provided and generally in terms of clinical outcomes.

## INTRODUCTION

The country of Mexico comprises 32 states, with an estimated total population in 2006 of 107,550,697 living in 2,454 municipalities^10^. Mexico has a relatively young population where about 32 percent are 14 years or younger and a further 19% are aged 15 to 24^22^. It has also a largely urban population where more than 76% of the population lives in urban areas^9^.

Mexico has a high prevalence of oral diseases with tooth decay affecting 61% of children over 6 years old^11^. Oral problems constitute the fifth most common reason for visits to the country's health services^16^. In terms of preventive programs for oral health, the Mexican Congress, as part of its 1989-1994 National Health Plan, declared that salt fluoridation should be one of the main priorities^12^. This followed on from the success of salt fluoridation trials initiated in 1973^6^. In 1991, Mexico became the seventh country in the world to adopt salt fluoridation to prevent dental caries^3^. The massive use of preventive measures including the use of fluoride toothpaste, education on prevention in the schools and the national program of salt fluoridation have resulted in the rapid decline in dental caries over the past two decades from a DMFT in 1989 of 4.4 for 12 year-olds^4^,^11^. The National Survey of Dental Caries in Mexico, conducted in 2001, reported that the prevalence of dental caries for schoolchildren aged 12 years was 58%, while the DMFT was 1.91. Of this the decayed tooth DT component was 1.54, missing teeth component MT 0.04, and the filled teeth component FT was 0.34^11^. This indicates that although the burden of dental caries in this age group has been substantially reduced through the use of fluoride, there remains a need for restorative treatment which is largely unmet.

### Steps leading up to the adoption of the ART approach in Mexico

The General Health Law of Mexico (Ley General de Salud)^12^ defines the powers for the establishment of national policies in the area of oral health. Chapter 45 paragraph 1 of the Internal Regulations of the Ministry of Health (Reglamento Interior de la Secretaría de Salud)^18^ details the need to propose policies for the prevention, treatment and control of oral disease. According to this regulation, the National Oral Health Program of Mexico defined and published a program of action for the years 2001-2006 (Programa de Acción: Salud Bucal 2001-2006) in which one of the strategies for improving oral health was to strengthen the curative care, expanding coverage to marginalized localities with problems of access and promote alternative curative treatment in the form of a countrywide adoption of the Atraumatic Restorative Treatment (ART) approach^16^.

The concept was to implement a plan for the introduction of the ART approach in public clinics in 19 states selected for their degree of marginalization and lack of access to care^11^. A number of barriers were however encountered. First, there was opposition to this approach by the dental association whose concerns included: a fear that caries would be left behind under ART restorations, that this in turn would lead to an irreversible pulpitis, and concerns about the reliability of the restorative material to be used. Other problems that emerged concerned the sourcing of suitable instruments and dental materials, especially high-strength glass ionomer, the availability of information material in Spanish on the ART approach and certain operational problems.

To resolve the latter problem a training manual in Spanish was published for national distribution in 2001^13^. This was followed by a organization of an international master training course on ART held in 2002 and attended by representatives of the Pan American Health Organization, the United States of America Air Force, Cayetano Heredia University of Peru and Caribbean countries, representatives of the 19 priority states, and representatives of the health and academic sector of Mexico.

Since then and up to the year 2006 there have been 27 theoretical and practical training courses where 810 dentists have been trained. In addition, a video on the clinical procedures involved in ART has been developed and is integrated into each ART training course. As a result of these initiatives the number of ART procedures provided has continued to increase from year to year. In 2000, a total of 177.823 ART procedures were reported to have been provided in government clinics rising to 712.869 in 2006. This represented an increase over this period of 400%^19^.

The National Development Plan (Plan Nacional de Desarrollo, 2007-2012)^14^ and the National Plan for Health 2007-2012 (Programa Nacional de Salud 2007-2012)^17^, have a number of strategies. The latter includes five main strategies:

1. To improve the health of the population; 2. To reduce gaps and inequalities in health through interventions targeted at vulnerable groups and marginalized communities; 3. To provide quality health and safety; 4. To prevent the impoverishment of the population for health reasons; and, 5. To ensure that health contributes to poverty reduction and social development of the country.

This incorporates the “100 Towns 100 Actions” strategy (“100 Municipios 100 Acciones”) which applies to the Municipalities which have the lowest Human Development Index (HDI) in the country^7^. It comprises a comprehensive strategy to fast track social development in these marginalized municipalities including increased housing supply, water and drainage, and projects for production.

A Specific Action Program for Oral Health, 2007-2012 (Programa de Acción Específico 2007-2012, Salud Bucal)^15^, outlines 13 strategic actions to improve oral health in Mexico. Strategic action number 9 is to extend the coverage of dental care through the use of Atraumatic Restorative Treatment in the 100 municipalities mentioned above. To achieve this goal, 19 additional ART courses were provided in 2008-2009 to a further 570 dentists, rising the total of number of dentists specifically trained in ART to 1380.

In parallel with the activities to train existing dentists about the ART approach, there have also been efforts to train dental students with the aim of improving their attitude to ART as an alternative treatment for carious lesions. This will help the newly trained dental graduate, during their obligatory (six months to one year) working in social service mainly in municipalities with a lower index of human development. Similarly, the Mexican dental associations of the country have also been invited to join this strategy.

### The attitudes of Mexican dentists to Atraumatic Restorative Treatment

**Figure 1:**
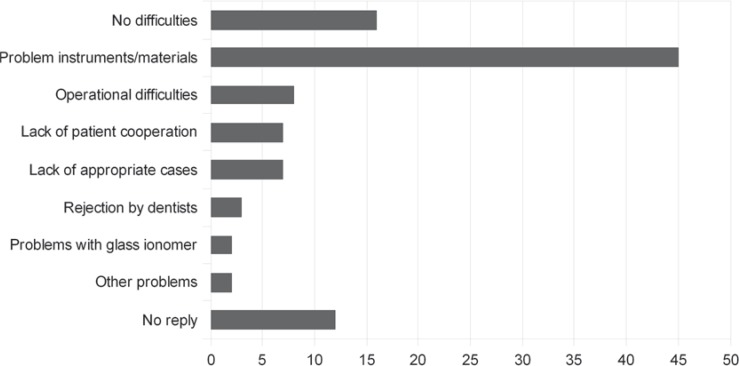
Major perceived difficulties when implementing the ART approach (percentage of responses)

In order to understand the attitude and views of Mexican dentists concerning the perceived major problems when implementing the ART approach in their practice and to determine where they considered such an approach could be best applied, a survey of 197 dentists was undertaken in 2008 in the states of Chiapas, Michoacan and Sinaloa. This survey found that the major problem for the implementation of ART, perceived by 45 % of the respondents, was the scarcity or unavailability of appropriate dental materials and the lack of suitable instruments; those that were available being of poor quality (Figure 1). It was also noted that the reason that dentists had joined ART training courses was not only to receive information on how to undertake ART correctly but also to facilitate access to appropriate materials and instruments.

This survey also identified that just over 55% of the dentists surveyed strictly followed the correct ART approach using hand instruments alone, while the remaining dentists used either a high- or low-speed handpiece either alone or to compliment the use of hand instruments when preparing a cavity for an “ART” restoration. The use of rotary instruments does not feature as part of the ART approach^2^, therefore the number of ART procedures reported from 2000-2006, given above, is most probably an overestimation. Since becoming aware of this reporting problem, the recording system for ART treatment has been corrected. In 2008 there were 241,449 ART treatments provided and from January to August 2009, a total of 172.815^19^.

### Preliminary evaluation of ART restorations and sealants provided in Mexico

The Specific Action Program for Oral Health, 2007-2012 (Programa de Acción específico 2007-2012, Salud Bucal)^15^ points to the need for surveillance of the oral health program for planning and decision making. This is achieved though the systematized monitoring mechanism that has been instrumental in following, for example, the number of ART procedures provided per year. While the number of procedures performed is one index of the success of the implementation of the ART approach in Mexico it does not provide information on specific outcomes relating to the implementation of the approach.

The National Oral Health Program in Mexico now has some nine years experience in using Atraumatic Restorative Treatment as one of its oral health strategies. Here it is considered to be an important alternative approach to the management of dental caries for marginalized areas of Mexico with problems of access, such as in those municipalities which have the lowest Human Development Index (HDI). It was therefore considered important to make a preliminary evaluation of ART restorations and sealants placed as part of this National Oral Health Program. A study was therefore designed to enable this to be undertaken. The primary objective was therefore to make a pilot evaluation after one year of ART restorations and sealants placed in primary and permanent teeth in schoolchildren aged 6 to 13 years. A secondary objective was to develop a tool for evaluating the effectiveness of the ART strategy in Mexico.

## MATERIAL AND METHODS

A prospective cohort study was conducted in 15 primary schools in 13 of the municipalities with the lowest Human Development Index (HDI) in six of the seven states in Mexico with such municipalities. A convenience sample of 304 schoolchildren aged 6 to 13 years was selected based on their need for one or more ART restorations or sealants. Informed consent was sought from the children's parents to participate in the study.

ART restorations were placed following standard ART procedures^1^ by 18 dentists who had been trained in the ART approach and who had prior experience with its use. All treatment was performed within the school facilities in areas either inside or outside the classroom. Only single-surface restorations were placed, that is, class I, III and V according to Black's classification. The same tooth could have a combination of ART treatment e.g. an ART sealant on the occlusal surface and a Class V restoration on the buccal surface. Ketac Molar Easymix (3M ESPE®) high-strength glass ionomer was used for all ART restorations and sealants.

**Figure 2:**
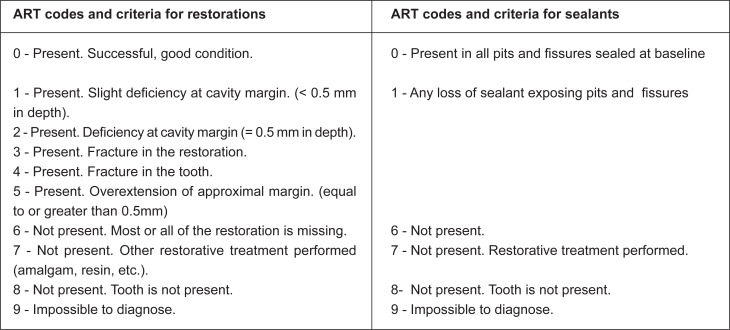
ART codes and criteria for restorations and sealants

**Figure 3:**
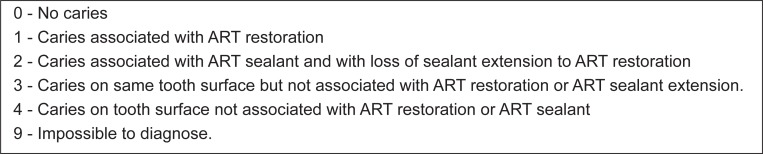
Criteria for diagnosing carious lesions in ART studies

The one-year evaluations were conducted by 7 examiners who had not been involved in the ART treatment and who had been trained by an external international expert over a four-day course. evaluations were undertaken using visual criteria alone with a plane mouth mirror and a WHO ball-ended periodontal probe. A specially designed form was used for registration and evaluation of the restorations and sealants. Standard ART criteria (Figure 2) was used to assess the ART restorations and sealants^20^. For this study, ART survival was measured by defining it as a restoration wear or not to submit this is not greater than 0.5mm. Caries was scored at the cavitational level (Figure 3). Furthermore, in two of the six states where the study took place, photographs of the ART treatment were taken for use in subsequent evaluations and for teaching purposes. Standard infection control procedures were observed for all examinations.

The examiner reproducibility was assessed using Kappa to be better than 0.82 for inter-examiner reproducibility and better than 0.92 for intra-examiner reproducibility.

Statistical analysis was undertaken using SPSS Software Version 15, Statistical Package.

## RESULTS

Of the 304 children who received ART treatment at baseline only 243 children were available at the one-year follow-up representing 80% of the original sample. In these children, 410 ART restorations were available for evaluation, 314 in primary teeth and 96 in permanent teeth. A total of 390 ART sealants were also evaluated, 182 in primary and 208 in permanent teeth.

The survival of ART restorations and those restorations associated with caries at the one-year evaluation for both primary and permanent teeth is given in Table 1. For this preliminary analysis the association between restoration failure and the finding of caries was not analyzed since this will be part of a subsequent reevaluation.

**Table 1:** Survival of ART restorations and those associated with caries at the one-year evaluation

	Number of ART restorations evaluated	Number of successful ART restorations at one year (%)	Number of ART restorations associated with caries (%)
Primary teeth	314	252 (80.2)	31 (9.8)
Permanent teeth	96	84 (87.5)	4 (4.1)
Overall	410	336 (81.9)	35 (8.5)

The survival of ART sealants at the one-year evaluation and caries associated with part-retained and totally lost sealants is given in Table 2. Since the number of previously sealed teeth with caries was very low, further statistical analysis was unwarranted.

**Table 2:** Survival of ART sealants and caries associated with partial or complete loss of sealant at the one-year evaluation

	Number of ART sealants evaluated	Number of fully or part-retained sealants at one-year (%)	Number of teeth with caries located in an area with part loss of sealant (%)	Number of teeth with caries located in an area with total loss of sealant (%)
Primary teeth	182	125 (68.7)	2 (1.6)	2 (3.5)
Permanent teeth	208	148 (71.2)	1 (0.7)	2 (3.3)
Overall	390	273 (70.0)	3 (1.1)	4 (3.4)

## DISCUSSION

This paper describes the process involved in introducing the ART approach in Mexico as part of an overall oral health strategy, a strategy which is firmly based on prevention with the emphasis on caries prevention. This has involved policy decisions at all levels of government culminating in the recent Specific Action Program for Oral Health, 2007-2012.

Six years after the introduction of ART as a strategy, in the year 2000, it was estimated that 2,750,899 separate ART procedures had been provided^19^. This is most likely to be an overestimation since the 2008 survey of Mexican dentists' attitudes to ART showed that some professionals had reported ART procedures even when they had used a low- or high-speed drill for cavity preparation. even when making a safe allowance for this overestimation, it still means that by the year 2006 well over a million ART procedures had been provided over this six-year period. This appoints to the huge success of the adoption of the ART approach strategy in Mexico. The ART strategy has also been progressively scaled up as more and more existing dentists are trained in the approach and as newly qualified dentists join the workforce having been trained during their university studies.

Since the Specific Action Program for Oral Health, 2007-2012^15^ ends in just over two years, it is only timely to undertake an evaluation of the outcomes of the strategy of using the ART approach as an important alternative approach to the management of dental caries for marginalized areas of Mexico with problems of access, such as in those municipalities which have the lowest Human Development Index (HDI). The evaluation of the ART program was not easy since it was spread over a number of Mexican states and this necessitated the use of a relatively large number of examiners for purely practical reasons. All the examiners did however follow a short training course with an international expert in an attempt to ensure consistency of results.

The survival results of this preliminary evaluation of ART restorations and sealants provided in the public service compare favorably with one other study conducted in Mexico by López, et al.^5^ (2005). In this two-year study, the acceptability and effectiveness of ART restorations and sealants for the prevention and treatment of dental caries were evaluated. A team of dentists and dental students from 2 dental schools and the Ministry of Health placed 370 ART restorations and 193 ART sealants in 118 subjects aged 5 to 18 years. eighty-five percent of subjects reported no pain and 93% reported being comfortable with their restorations. The subjects were assessed at 1 and 2 years, showing an overall retention of ART restorations in permanent teeth of 81% and 66% respectively. This is poorer than the 87.5% survival reported in this present study at one year for similar restorations. The retention of ART sealants in the Lopez study was also poorer with a one year survival of 51% against the 71.2% found in this present study. It is not clear why this should be the case.

While the results of this present study are encouraging they fall short of the survival results reported in a meta-analysis of studies published up to 2005 where weighted mean survival for one-surface ART restorations were 97% for permanent teeth and 95% for primary teeth^21^. Similarly, although the number of ART sealant papers is limited in number, weighted mean survival rates in the region of 90% after one year have been reported. The reasons for lower survival rates in the present study will need to be explored but there are a number of possible reasons that might explain this. The outcomes could be due to the dentists failing to select suitable teeth for ART restorations and sealants, or though them failing to rigidly follow the ART treatment protocol. The relatively high percentage of failure due to caries with ART restorations, which hasn't been reported in other ART studies, needs further examination. Here, the photographs which were taken will be very useful in the future to identify whether this could have been the case but also as a teaching tool both in ART courses and in ART calibration training sessions. Moreover, the two-year evaluation will be decisive in shedding light on these matters so firm conclusions on the survival outcomes of ART restorations and sealants placed in the Mexican public health service.

Notwithstanding the survival outcomes, the one-year evaluation can be considered to be a operational success since it has shown that it is possible to evaluate a public program where ART restorations and sealants are being provided even though this might cover a large number of geographical remote areas, in this case a number of Mexican states. Likewise, the specially designed form for recording and monitoring ART treatment was convenient and easy to use, and might be scaled up for monitoring activities on a daily basis within the country's health services.

## CONCLUSIONS

Mexico has an ambitious and forward thinking policy for improving the oral health of its population. The results from the evaluation of the ART strategy show that it has been successful both in terms of the number of ART procedures performed since its introduction and generally in terms of clinical outcomes. This evaluation has also been useful in identifying areas where improvements could be made as part of the strategically planning cycle. While in this publication we have concentrated on the ART strategy in Mexico, it is important to reiterate that ART is just one component of Mexico's overall oral health strategy firmly based on prevention and improving access to care countrywide.
